# Tocotrienol-Rich Vitamin E from Palm Oil (Tocovid) and Its Effects in Diabetes and Diabetic Nephropathy: A Pilot Phase II Clinical Trial

**DOI:** 10.3390/nu10091315

**Published:** 2018-09-17

**Authors:** Suzanne May Quinn Tan, Yilynn Chiew, Badariah Ahmad, Khalid Abdul Kadir

**Affiliations:** School of Medicine and Health Sciences, Monash University Malaysia, Selangor 46150, Malaysia; drchiewyilynn@gmail.com (Y.C.); badariah.ahmad@monash.edu (B.A.); khalid.kadir@monash.edu (K.A.K.)

**Keywords:** advanced glycation end products, carboxymethyl-lysine, tocotrienol, vitamin E, antioxidant, diabetes, diabetic nephropathy, oxidative stress

## Abstract

Tocotrienol-rich vitamin E from palm oil (Tocovid) has been shown to ameliorate diabetes through its superior antioxidant, antihyperglycemic, and anti-inflammatory properties in diabetic rats. This study aimed to investigate the effects of Tocovid on diabetic nephropathy in patients with type 2 diabetes. Baseline parameters of potential subjects such as HbA1c, blood pressure, Advanced Glycation Endproduct (AGE), soluble receptor for AGE (sRAGE), Nε-Carboxymethyllysine (Nε-CML), and Cystatin C were assessed for possible correlation with diabetic nephropathy. Only subjects with diabetic nephropathy or urine microalbuminuria-positive defined as Urine Albumin to Creatinine Ratio (UACR) >10 mg/mmol were recruited into a prospective, randomized, double-blinded, placebo-controlled trial. The intervention group (*n* = 22) received Tocovid 200 mg twice a day while the control group (*n* = 23) received placebo twice a day for 8 weeks. Changes in Hemoglobin A1c (HbA1c), blood pressure, serum biomarkers and renal parameters such as UACR, serum creatinine, and estimated Glomerular Filtration Rate (eGFR) were compared between the two groups. It was found that serum Nε-CML significantly correlated to the severity of microalbuminuria. For every 1 ng/mL increase in serum Nε-CML, the odds of diabetic nephropathy increased by 1.476 times. Tocovid, compared to placebo, significantly reduced serum creatinine but not eGFR, UACR, HbA1c, blood pressure, and serum biomarkers. In conclusion, serum Nε-CML is a potential biomarker for diabetic nephropathy. Treatment with Tocovid significantly reduced serum creatinine; therefore Tocovid may be a useful addition to the current treatment for diabetic nephropathy.

## 1. Introduction

Diabetes is a major global health crisis of the 21st century. The number of people affected by diabetes quadrupled from 108 million people in 1980 to a staggering figure of 422 million people worldwide in 2014 [1]. One of the major complications of diabetes is diabetic nephropathy, the leading cause of end stage renal failure (ESRF) worldwide. Costly renal replacement therapies, such as dialysis or kidney transplant, are needed in order for patients with ESRF to survive [2]. ESRF has affected 2 million people globally, with a rapid growth rate of approximately 5–7% per year [3]. Furthermore, patients with diabetic nephropathy have increased risk of cardiovascular disease (CVD) events, hospitalizations [4], cognitive dysfunction [5], and poor quality of life [6,7]. Therefore, treatment to prevent the onset and progression of diabetic nephropathy is vital.

Vitamin E is a potent antioxidant capable of reducing oxidative stress and inflammation, the key players in the pathogenesis of diabetic complications [8]. The two major constituents of vitamin E are tocopherol and tocotrienol. Tocotrienol has been proven to be 40 to 60 times more potent antioxidant compared to tocopherol with superior antiglycemic, anticholesterolemic, anti-inflammatory, neuroprotective, and cardioprotective properties [9]. However, the renoprotective effect of tocotrienol in type 2 diabetes is currently limited, and has not been clinically investigated. The MICRO-HOPE study reported that 4.5 years of vitamin E at a dose of 400 IU did not have any significant effect on the risk and progression of diabetic nephropathy in patients with type 2 diabetes [10]. This was further supported in other studies, systemic reviews, and meta-analysis [10]. However, these studies were based on tocopherol-rich vitamin E, and not tocotrienol-rich vitamin E. Tocotrienol consisted of merely 3% of all research studies on vitamin E in PubMed. Out of 927 clinical trials on vitamin E, only 25 trials were on tocotrienol, and none of them investigated the effect of tocotrienol in diabetic nephropathy [9].

Nevertheless, results from preclinical studies on tocotrienol and diabetic nephropathy have been promising. Tocotrienol-rich fraction attenuated lipid-induced nephropathy in diabetic rats through its antihyperglycemic, anticholesterolemic, and renoprotective effects. The enhanced renal function was achieved through downregulation of inflammatory and profibrotic cytokines such as transforming growth factor beta-1 (TGF-β1), tumor necrosis factor-α (TNF-α), and nuclear factor kappa-light-chain-enhancer of activated B cells (NF-kB) [11,12]. The current treatment, which is strict glucose and blood pressure control, does not address these inflammatory pathways [12]. Therefore, tocotrienol-rich vitamin E (Tocovid) was proposed as an addition to the current treatment for diabetic nephropathy.

Besides that, Tocovid is capable of reducing advanced glycation end product (AGE), a toxic by-product of glucose, protein, or lipid oxidation [13,14]. AGE, a novel biomarker, can cause oxidative stress by binding to its receptor (RAGE). Formation of AGE is accelerated under hyperglycemic states, and can accumulate in the body despite normalization of HbA1c [14,15]. Although reduction of HbA1c resulted in short-term improvement in diabetic nephropathy [16,17], more recent studies, such as the follow-up studies of Diabetes Control and Complications Trial/Epidemiology of Diabetes Interventions and Complications (DCCT/EDIC) study and the United Kingdom Prospective Diabetes Study (UKPDS), showed that HbA1c did not correlate well with diabetic nephropathy in the long run [18,19]. This was because HbA1c lasts for only 3 months in the body, whereas AGE lasts a lifetime. Studies have correlated AGE levels in the serum and tissues to the development of diabetic nephropathy [20,21]. Therefore, the reduction of AGE, and not HbA1c, may prevent the onset and progression of diabetic nephropathy in the long run.

One of the well-characterized types of AGE in humans is Nε-CML. In the DCCT-EDIC study, Nε-CML in skin collagen was found to predict the risk of future 10-year progression of diabetic nephropathy [22]. In contrast to RAGE, the soluble form of RAGE (sRAGE) neutralizes the effects and signals of RAGE, leading to the prevention of oxidative stress [23]. Furthermore, the ratio of serum AGE to the sRAGE was found to be an independent predictor of endothelial dysfunction [24]. On the other hand, Cystatin C is a novel marker of renal function shown to be superior to serum creatinine in eGFR estimation [25].

The present study aimed to establish a correlation between diabetic nephropathy with HbA1c, blood pressure, AGE, sRAGE, Nε-CML, and Cystatin C. The second aim is to investigate whether high-dose Tocovid will have an effect on diabetic nephropathy assessed by UACR, serum creatinine, and eGFR. Then, this effect will be examined for any correlation to changes in HbA1c, blood pressure, AGE, sRAGE, Nε-CML, and Cystatin C.

## 2. Materials and Methods

### 2.1. Participants and Study Enrolment

Participants were recruited from an existing pool of patients who come for regular follow-ups at the Monash University Clinical Research Center (CRC) in Bandar Sunway and in Johor Bahru. The participants were selected to come for screening if they fulfilled the inclusion and exclusion criteria based on their past records at the research centers. Participants who were interested in the study were reminded, over the phone, to come in after fasting for at least 8 h, as per any routine diabetes follow-ups, to avoid strenuous exercise the night before, and to skip their morning dose of anti-diabetic medications. Premenopausal female participants were reminded to come for screening during their non-menstrual period. Participants who had fallen ill before their screening visit were allowed to fully recover before they joined the study.

Informed consent was obtained from all participants before the screening began. The study was carried out in accordance with the Declaration of Helsinki, and the study protocol was approved by the Monash University Human Research Ethics Committee (Project number: 12090). Complete history-taking and physical examination were conducted during screening. Weight and height were recorded twice without wearing shoes. Waist circumference was also measured by aligning the bottom edge of the measuring tape with the top of the hip bone and, then, wrapping the tape measure all the way around the waist. The reading was recorded twice during exhalation.

The participants’ blood pressure was measured three times on the left arm in a sitting position, and an average of three stable readings was taken. Other screening tests included were fasting blood glucose, HbA1c, urine dipstick test, UACR, and urine pregnancy test for premenopausal women. Safety blood tests, such as renal profile, liver function test, lipid profile, and ECG were conducted to ensure that the participants are fit to participate.

### 2.2. Preliminary Assessment

Participants must be aged between 18 to 80 years old, and diagnosed with type 2 diabetes with stable glucose control (not more than 10% change over the last 2 months) in order to participate. If the participants have hypertension, their blood pressure control must be stable (not more than 10% change over the last 2 months), and the range should be less than 160/100. A microalbuminuria-positive result in diabetic patients must be due to diabetic nephropathy alone. Therefore, the exclusion criteria were the presence of known non-diabetic kidney disease (i.e., kidney stones, minimal change disease, or idiopathic membranous nephropathy, IgA nephropathy, and acute tubulointerstitial nephritis) or untreated urinary tract infection or menstrual period during screening. This is crucial because these presentations can cause a falsely high UACR level that is not due to diabetic nephropathy. Another exclusion criterion was the presence of acute or severe chronic illnesses, such as acute coronary syndrome, active tuberculosis, active cancer, liver, or inflammatory diseases. Participants who were pregnant were also excluded.

Baseline characteristics of participants who met the inclusion and exclusion criteria during the preliminary assessment were recorded for analysis. Serum AGE, sRAGE, Nε-CML and Cystatin C from eligible participants were analyzed.

### 2.3. Randomized Controlled Trial (RCT)

Participants who passed the preliminary assessment were further assessed for eligibility into a prospective, randomized, double-blinded, placebo-controlled clinical trial. Participants must have diabetic nephropathy or UACR ≥10 mg/mmol (3–4 times above the upper limit of normal) in order to join the trial. The normal UACR range for females is ≤3.5 mg/mmol and, for males, is ≤2.5 mg/mmol. Besides that, participants are excluded if they have consumed water-soluble antioxidants, such as polyphenols, glutathione, vitamin B or C, during the past 2 weeks, or fat-soluble antioxidants such as vitamin A, D, E, and K during the past 1 month. This is to ensure that the outcome of the trial is due to the intervention alone, and not because of other antioxidants.

The intervention group received 200 mg twice a day Tocotrienol-rich vitamin E (Tocovid Suprabio^TM^) (Hovid Berhad, Ipoh, Malaysia), while the control group was given placebo twice a day for eight weeks. The ingredient of the Tocovid Suprabio^TM^, called EVNol SupraBio was manufactured by ExcelVite, Malaysia. The high dosage of Tocovid was used because previous clinical studies showed that Tocovid at low doses did not yield significant results [26]. The dose selected is the maximum dose approved by the Food and Drug Administration (FDA). The study duration of eight weeks was based on previous studies that reported eight weeks of Tocovid had successfully reduced lipid peroxidation in humans [26] and AGE, HbA1c, and RAGE expression in the liver of rats with metabolic syndrome [13].

### 2.4. Sample Size

The power calculations were based on the ability to detect a 30% reduction in UACR in the primary analysis of Tocovid compared to placebo, assuming a 5% SD of effect (α = 0.05 and β-1 = 0.8) and an anticipated dropout rate of 4%. To fulfill these specifications, 52 subjects were required.

### 2.5. Randomization

Randomization was conducted in a 1:1 ratio by an independent consultant who provided the computer-generated random allocation sequence with permuted blocks of size 4, stratified according to age, gender, and duration of diabetes. Treatment allocation was concealed using sequentially numbered opaque sealed envelopes. Participants and principal investigators were blinded to the allocation into study groups. The capsules of both placebo and Tocovid were similar in size, shape, and excipients. The identity of the study drugs was kept confidential by ExcelVite and they were labeled as Drug A and Drug B.

### 2.6. Follow-Up Visits

Monthly follow-ups were carried out to monitor for adverse drug events and compliance to treatment. Participants were asked to return their drug bottles for capsule count in every follow-up to ensure compliance. Anthropometric measurements, blood pressure, finger-prick test, and urine dipstick were routinely carried out in every visit for monitoring purposes. In the final visit, the participants’ HbA1c, serum creatinine, eGFR, UACR, and serum biomarkers were repeated. Safety blood tests were also repeated and compared to baseline to ensure that the study drugs had no adverse effects on the vital organs such as liver, kidneys, and heart.

**Figure 1:**
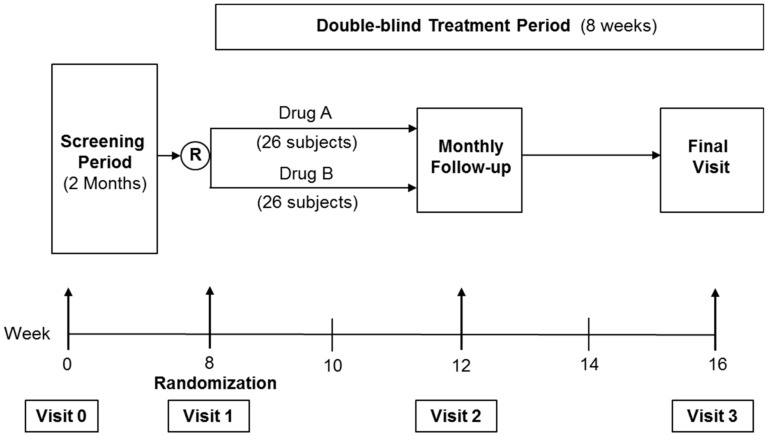
Overall study design.

The identity of the study drugs were revealed by ExcelVite at the end of the study. Drug A was Tocovid while drug B was placebo. The overall study design is as shown in Figure 1.

### 2.7. Assessment of Outcomes

The primary outcome variables of this study were HbA1c, blood pressure, AGE, sRAGE, Nε-CML, and Cystatin C. The secondary outcome variables were the renal parameters; UACR, serum creatinine, and eGFR.

### 2.8. HbA1c

Determination of HbA1c is a useful test to measure glycemic control over the past three months. Only 1.5 µL of capillary blood sample was required to run the test. The HbA1c kit (Alere Afinion, Waltham, MA, USA) has a measuring range of 4–15% with coefficient variances (CV) of less than 3%.

### 2.9. BUSE Creatinine, Lipid Profile, and Liver Function Test

During the screening and final visit, 18 mL of fasting blood samples were taken and collected into two serum-separating tubes (SST) from each participant. To separate the serum, the blood samples were centrifuged (Eppendorf Centrifuge 5702R, Hamburg, Germany) at 3600 rpm for 15 min. Subsequently, the serum samples were extracted and transferred into 1 mL Eppendorf tubes. Some of the serum samples were sent to a national certified pathology lab to be tested for serum creatinine, blood urea nitrogen (BUN), lipid profile, and liver function test (Abbott diagnostic ARCHITECT, Abbott Park, IL, USA) on the same working day. The coefficient variances for these tests were generally less than 6%.

### 2.10. AGE, sRAGE, Nε-CML, and Cystatin C

Serum samples for AGE, sRAGE, Nε-CML, and Cystatin C were stored in a −80 °C fridge. Processing of the biomarkers was done at the end of study, on a batch-to-batch basis, to minimize inter-assay variation. Serum AGE, sRAGE, Nε-CML, and Cystatin C concentrations were measured in duplicates and quantified by calorimetric method using Enzyme-linked Immunosorbent Assay (ELISA) (TECAN Infinite 200 PRO, Männedorf, Zürich, Switzerland). The ELISA kit for AGE (Cell Biolabs STA-317, San Diego, CA, USA), Nε-CML (Cell Biolabs STA-816, San Diego, CA, USA), sRAGE (Elabscience E-EL-H0295, Houston, TX, USA), and Cystatin C (Elabscience E-EL-H0055, Houston, TX, USA) had intra-assay coefficient variances of 4% and inter-assay coefficient variances of 8%.

### 2.11. UACR

The urine samples were sent to the national pathology lab on the same working day to test for UACR. The UACR kit (Abbott diagnostic ARCHITECT, Abbott Park, IL, USA) had coefficient variances of ≤6% for microalbuminuria and ≤5% for urine creatinine. Two UACR readings were taken on two different days (screening visit and visit 1) to obtain the average UACR level at baseline. Both readings must be ≥10 mg/mmol in each subject. UACR is a very accurate test of proteinuria because it is unaffected by the variation of urine concentration, unlike a urine dipstick test. The UACR readings were classified into several grades (0–29, 30–149, 150–299, and ≥300 mg/mmol) to reflect the severity of microalbuminuria in patients with type 2 diabetes.

### 2.12. Statistical Analysis

All statistical analyses were conducted using Statistical Package for Social Sciences (SPSS) version 25 (IBM SPSS Inc, Chicago, IL, USA). Baseline characteristics of study participants were presented using chi-square test for categorical variables and independent *t*-test for numerical variables.

During the preliminary assessment, baseline parameters such as HbA1c, blood pressure, and serum biomarkers were compared between several UACR grades using analysis of covariance (ANCOVA) with post hoc Bonferroni test for pairwise comparisons. A partial correlation test was applied to correlate baseline parameters with serum creatinine and eGFR while controlling for confounding factors. Univariate and multivariate logistic regression were conducted to determine the odds of diabetic nephropathy assessed by UACR in relation to the baseline parameters. Model fit assessment was done using Hosmer–Lemeshow test, classification table, and area under receiver operating characteristics (ROC) curve.

In the RCT, assessment of analytes at baseline between placebo and Tocovid group was conducted using an independent *t*-test. Treatment changes at the end of the study were compared between both groups using ANCOVA, adjusting for the respective baseline values. *p*-values < 0.05 were deemed statistically significant.

## 3. Results

A total of 118 type 2 diabetic patients were screened over 2 months. Of these, 59 subjects did not meet the inclusion criteria and were excluded. Another three male subjects were excluded because of kidney stones that caused hematuria. Three female subjects had microalbuminuria but tested positive for urinary tract infection (UTI). They were treated with cefuroxime 250 mg BD for 1 week, and then returned for a repeat screening visit. Dipstick test for all three women showed no UTI after treatment, but their microalbuminuria remained positive, thus, they were included in the study. None of the premenopausal women had their menstrual period during the screening. In total, 66 patients were eligible after the preliminary assessment, and their baseline parameters were recorded.

**Figure 2:**
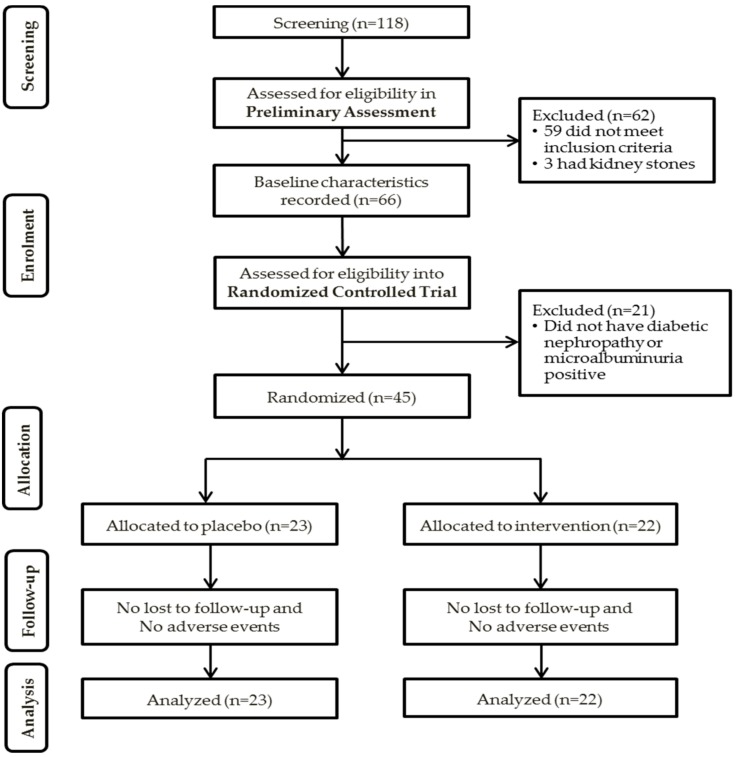
Summary of patient flow diagram. n: number of participants.

In the RCT, 21 of the 66 patients were excluded because they did not have diabetic nephropathy or were microalbuminuria-positive (UACR >10 mg/mmol). Five subjects were on antioxidants during screening, and they were asked to stop their antioxidant intake for at least two weeks (if water-soluble antioxidant) or four weeks (if fat-soluble antioxidant) before returning for the randomization visit. A total of 45 patients were included into the RCT; 22 in the intervention group and 23 in the placebo group. There were no adverse events and none were lost upon follow-up. The summary of the patient recruitment flowchart is shown in Figure 2.

### 3.1. Preliminary Assessment

The baseline characteristics of the 66 participants who passed the preliminary assessment are shown in Table 1. The participants were predominantly males and of Malay ethnicity. The mean age was 61.6 years, and the mean duration of diabetes was 18.5 years. The average HbA1c reading was 8.9%, while the average systolic blood pressure (SBP) and diastolic blood pressure (DBP) readings were 136.7 mmHg and 77.2 mmHg, respectively. The mean Body Mass Index (BMI) was 29 kg/m^2^, which is considered as overweight or pre-obese by World Health Organization (WHO) [27].

#### 3.1.1. Correlations with UACR Grades

There was a significant difference in Nε-CML between the UACR grades, despite controlling for potential confounding factors such as HbA1c, SBP, DBP, age, and duration of diabetes (Table 2). Post hoc Bonferroni test showed that the mean Nε-CML in UACR grade 30–149 mg/mmol was significantly higher than the mean Nε-CML in UACR grade 0–29 mg/mmol (*p =* 0.049). This showed that serum Nε-CML was significantly raised in subjects with diabetic nephropathy compared to those without.

However, there were no significant differences in AGE, sRAGE, and Cystatin C between the UACR grades (Table 2). Similarly, there was no significant difference in HbA1c, systolic blood pressure (SBP), diastolic blood pressure (DBP), age, and duration of diabetes between the UACR grades, as shown in Table 3.

#### 3.1.2. Correlations with Serum Creatinine and eGFR

Based on Table 4, serum Nε-CML showed a significant correlation with serum creatinine and eGFR despite controlling for HbA1c, SBP, DBP, and age. Nε-CML had a low positive correlation with serum creatinine and a low negative correlation with eGFR. There were no significant correlations in the rest of the parameters, even after controlling for confounding factors.

#### 3.1.3. Correlations with Nε-CML

Nε-CML had a significant moderate positive correlation with AGE after controlling for HbA1c and age, as shown in Table 5. There were no significant correlations between Nε-CML and the other serum biomarkers, HbA1c, age, and duration of diabetes.

#### 3.1.4. Univariate and Multivariate Analysis

Binary logistic regression was used to determine the odds of diabetic nephropathy in relation to the HbA1c, SBP, DBP, AGE, sRAGE, Nε-CML, and Cystatin C levels. Upon univariate analysis, there was a significant relationship between diabetic nephropathy and DBP, AGE, sRAGE, Nε-CML, and Cystatin C (*p* < 0.250), as shown in Table 6.

On multivariate analysis, only serum Nε-CML showed statistical significance with diabetic nephropathy (*p* < 0.05). For every 1 ng/mL increase in serum Nε-CML, the odds of diabetic nephropathy increased by 1.476 times (*p* = 0.008).

Model fit assessment was conducted for the multiple logistic regression model using three methods: Hosmer–Lemeshow test, classification table, and area under receiver operating characteristics (ROC) curve. In the Hosmer–Lemeshow test, the *p*-value was above 0.05 (*p* = 0.40), indicating a good model fit to data. In the classification table, the percentage which is correctly classified is expected to be more than 70% for a good model fit. In this model, 72.1% of the subjects were correctly classified by the model. The area under ROC curve (AUC) is an acceptable fit if >0.7. In this model, the AUC was 72.1% (95% CI: 0.595, 0.828).

### 3.2. Randomized Controlled Trial

General characteristics of the participants included in the trial are presented in Table 7. There were no significant differences in sociodemographic characteristics and baseline analytes, such as HbA1c, SBP, DBP, weight, BMI, renal parameters, lipid profile, and serum biomarkers between the placebo and Tocovid group. For the liver function test, the intervention group had a significantly higher aspartate aminotransferase (AST) and alanine aminotransferase (ALT) levels compared to the placebo group, but they were within the normal range.

Table 8 compared the treatment changes between the placebo and Tocovid group at the end of eight weeks. Tocovid significantly reduced serum creatinine compared to placebo (−11.28 umol/L ± 4.31, *p* = 0.014) despite adjustment for baseline value of serum creatinine, HbA1c, SBP, DBP, age, weight, AGE, CML, sRAGE, and Cystatin C. The result suggests that Tocovid prevented the progression of renal impairment by stabilizing serum creatinine when compared to placebo. There were no significant changes in the rest of the analytes, even after adjustment for their baseline values respectively. The compliance rate was 97% in the placebo group and 98% in the Tocovid group.

## 4. Discussion

This study showed that diabetic nephropathy was significantly associated with serum Nε-CML and not the conventional risk factors, such as hyperglycemia, assessed by HbA1c and blood pressure. This is a new finding, as there are no other studies that have demonstrated a significant correlation between serum Nε-CML and diabetic nephropathy that is independent of HbA1c, blood pressure, age, and duration of diabetes. In fact, serum Nε-CML was significantly correlated to diabetic nephropathy in patients with type 1 diabetes, but not type 2 diabetes [28,29,30]. Although the pathophysiology of type 1 and type 2 diabetes are inherently different, the development of diabetic complications in type 1 and type 2 diabetes are due to same root cause, which is increased oxidative stress causing excessive production of AGEs, such as Nε-CML [29,31]. Therefore, Nε-CML should correlate with the progression of diabetic nephropathy in both type 1 and type 2 diabetes.

Nε-CML is a type of AGE that accumulates in protein tissues and increases with age, but in diabetes, it increases at an accelerated rate. This similarity in origin explains the strong correlation between Nε-CML and AGE in this study (Table 5). Nε-CML was initially thought to be a by-product of non-enzymatic glycation of plasma proteins leading to formation of toxic Amadori products on protein. However, studies have now shown that Nε-CML is also derived from polyunsaturated fatty acid (PUFA) during lipid peroxidation, independent of the presence of Amadori products on protein. In fact, lipid peroxidation was found to be the main source of Nε-CML because PUFAs generally undergo autoxidation more easily compared to carbohydrates, even during a chronic hyperglycemia state, where the concentrations of glucose and Amadori products on protein are elevated.

In this study, the AGE conjugate used for ELISA assay is a pure protein-based advanced glycation end product, because it is derived from albumin only. On the other hand, Nε-CML is a by-product of both protein glycation as well as lipid peroxidation, causing it to be a more complex and toxic oxidative product compared to albumin-based AGE [32]. Since diabetic nephropathy was strongly associated with Nε-CML and not albumin-based AGE, the pathogenesis of diabetic nephropathy may be predominantly related to lipid peroxidation, rather the protein glycation.

This study also showed that Nε-CML was significantly correlated to renal function assessed by serum creatinine and eGFR levels, despite controlling for HbA1c, blood pressure, and age (Table 4). This result was in agreement with other studies reporting that serum Nε-CML had a direct correlation with serum creatinine in diabetic patients with reduced eGFR. This is because the onset of renal impairment impairs the function of the kidneys to remove serum creatinine and Nε-CML through urinary excretion. This was shown in patients with end-stage renal failure who had extremely high levels of serum Nε-CML (increased three to fourfold compare to healthy subjects) [28]. Hence, Nε-CML was associated with all renal parameters of diabetic nephropathy in this study.

Besides that, serum Nε-CML may be a better marker than UACR because it is directly related to oxidative stress in the pathogenesis of diabetic nephropathy, whereas UACR can be falsely high due to numerous confounding factors, such as kidney stones, menstrual period, or urinary tract infection. Furthermore, Nε-CML could be used in the prevention of diabetic nephropathy given its ability to detect the odds of diabetic nephropathy at an early stage. Nε-CML could also be used to monitor for response to therapy in patients with diabetic nephropathy. Therefore, the clinical relevance of Nε-CML is very high and potentially rewarding. Nevertheless, future studies are needed to determine the accuracy of serum Nε-CML in reflecting the odds and progression of diabetic nephropathy.

In the randomized controlled clinical trial, high-dose Tocovid for 8 weeks did not decrease HbA1c, blood pressure, serum AGE, RAGE, Nε-CML, and Cystatin C compared to placebo. These findings were contrary to previous studies that showed 8 weeks of Tocovid significantly reduced HbA1c, blood pressure, AGE, and RAGE expression in the liver of rats with metabolic syndrome [13]. The lack of significant effects may be attributed to the long duration of diabetes among the study participants, causing a long-term accumulation of AGE and Nε-CML that persisted in the body despite receiving Tocovid at a high dose. Studies have shown that AGE-modified collagens are hard to degrade, and can remain in diabetic vessels, kidneys, and the heart for a prolonged period of time [14]. The mean duration of diabetes in this study was about 18 years.

Similarly, the participants with long-standing type 2 diabetes in the Veterans Affairs Diabetes Trial (VADT), Action to Control Cardiovascular Risk in Diabetes (ACCORD), and Action in Diabetes and Vascular Disease: Preterax and Diamicron Modified Release Controlled Evaluation (ADVANCE) studies had no significant reduction in the risk of diabetic complications, despite a reduction in HbA1c through intensive glycemic control [33,34,35]. In fact, the mortality rate in the treatment group increased by 22%, causing the ACCORD study to terminate early [34]. The long-term accumulation of AGE among the participants in these studies may have hindered the reduction in the risk of diabetic complications, because AGE can persist despite normalization of HbA1c causing damage to the organs deposited with AGE [14]. The mean duration of diabetes in the ADVANCE, ACCORD, and VADT trials was about 8, 10, and 12 years, respectively [36].

On the other hand, the UKPDS and DCCT studies showed that newly diagnosed diabetic patients had a significant reduction in the risk of diabetic nephropathy, retinopathy, and clinical neuropathy when treated with an intensive glucose control [16,17]. Similarly, in newly induced diabetic rats, 16 weeks of Tocovid had not only reduced hyperglycemia, HbA1c, and lipid peroxidation, but also reversed lipid-induced nephropathy [11]. The short duration of diabetes in these studies may be the key factor because the amelioration of hyperglycemia reduces oxidative stress which, in turn, decreases the formation of AGE. In the long run, this would prevent the accumulation of AGE and the long-term damage to the tissues and organs in the body, thus preventing the onset of diabetic complications [15].

Nevertheless, Tocovid significantly reduced serum creatinine compared to placebo, indicating a different pathway was used by Tocovid to reduce serum creatinine. This pathway may implicate inflammatory biomarkers with shorter half-life, such as interleukin-6, TGF-β, and TNF-α, because studies have shown that Tocovid reduced serum creatinine and ameliorated diabetic nephropathy through downregulation of TGF-β and TNF-α in diabetic rats [12]. Future clinical trials are warranted to investigate this pathway. Previous animal studies showed that 11 weeks of tocotrienol-rich fraction extracted from palm and rice brain oils significantly improved the creatinine clearance. This effect persisted until the end of the study at 16 weeks [11]. Perhaps, if this study were to continue for a longer period of time, there would be a significant improvement in eGFR levels in patients treated with Tocovid compared to placebo.

One of the main limitations of this study was the small sample size. The ideal sample size for this clinical trial was 52 participants but only 45 subjects were recruited. As a result, the statistical power of this study decreased, making it difficult to detect any significant changes or effects of Tocovid compared to placebo. The next limitation is the short study duration. Considering that AGE, sRAGE, and Nε-CML are long-lasting biomarkers, eight weeks of Tocovid was probably insufficient to observe any noticeable reduction in AGE, sRAGE, and Nε-CML, even at a very high dose of 200 mg BD. In future, a longer study duration with larger sample size is needed. Another limitation of this study is the lack of newly diagnosed diabetic patients. This was because the study intended to recruit patients with diabetic nephropathy. Like any other diabetic complications, diabetic nephropathy takes many years to develop, thus, it is expected that the duration of diabetes among the participants was long. Nevertheless, future studies should investigate the potential role of Tocovid in preventing diabetic complications among patients who are newly diagnosed with type 2 diabetes. Lastly, future studies should include measuring of serum vitamin E to investigate for confounding effects.

## 5. Conclusions

In conclusion, diabetic nephropathy was significantly associated with serum Nε-CML, which highlights the potential clinical use of Nε-CML in assessing the odds of diabetic nephropathy at an early stage. Eight weeks of high-dose Tocovid did not improve HbA1c, blood pressure, serum AGE, sRAGE, Nε-CML, and Cystatin C in patients with diabetic nephropathy. Nevertheless, Tocovid significantly reduced serum creatinine compared to placebo. Therefore, Tocovid may be a useful addition to the current treatment for diabetic nephropathy.

## Figures and Tables

**Table 1:** Baseline characteristics of 66 patients with type 2 diabetes.

Baseline Characteristics	Participants (*N* = 66)
Gender, *n* (%)	
Male	48 (72.7)
Female	18 (27.3)
Race, *n* (%)	
Malay	34 (51.5)
Chinese	14 (21.2)
Indian	18 (27.3)
Age (years) *	61.6 ± 9.5
Duration of DM (years) *	18.5 ± 8.9
HbA1c (%) *	8.9 ± 1.7
SBP (mmHg) *	136.7 ± 15.3
DBP (mmHg) *	77.2 ± 9.1
BMI (kg/m^2^) *	29 ± 4.8

* Data presented as means ± standard deviations. N: Number of participants, DM: diabetes mellitus, HbA1c: Hemoglobin A1c, SBP: Systolic Blood Pressure, DBP: Diastolic Blood Pressure, BMI: Body Mass Index.

**Table 2:** Comparison of serum biomarkers between urine microalbuminuria (UACR) grades.

UACR Grade (mg/mmol)	*N* (%)	AGE (µg/mL)	sRAGE (pg/mL)	Nε-CML *^,†^ (ng/mL)	Cystatin C (ng/mL)
0–29	29 (43.9)	61.4 ± 95.8	956.5 ± 467.8	1.46 ± 0.36	2235.1 ± 956.7
30–149	29 (43.9)	159.3 ± 191.6	1131.9 ± 395.4	2.85 ± 0.36	1933.2 ± 906.8
150–299	5 (7.6)	90.9 ± 65.3	1301.9 ± 643.8	1.92 ± 0.90	1767.7 ± 1114.9
≥300	3 (4.5)	12.7 ± 9.9	1020.6 ± 443.4	3.41 ± 1.10	2043.8 ± 928.2

All values are presented as means ± standard errors of the mean. ^†^ Post hoc Bonferroni test applied. * Significant at *p* < 0.05. UACR: Urine Albumin to Creatinine Ratio, N: Number of participants, AGE: Advanced Glycation Endproduct, sRAGE: soluble Receptor for AGE, Nε-CML: Nε-Carboxymethyllysine.

**Table 3:** Comparison of HbA1c, systolic blood pressure, diastolic blood pressure, age, and duration of diabetes between urine microalbuminuria (UACR) grades.

UACR Grade (mg/mmol)	*N* (%)	HbA1c (%)	SBP (mmHg)	DBP (mmHg)	Age (years)	Duration of Diabetes (years)
0–29	29 (43.9)	8.7 ± 1.6	136 ± 11	76 ± 8	62.5 ± 7.8	17.8 ± 9.4
30–149	29 (43.9)	8.9 ± 1.9	136 ± 18	79 ± 10	61.7 ± 10.4	18.8 ± 8.6
150–299	5 (7.6)	10.2 ± 2.2	140 ± 23	75 ± 8	54.6 ± 14.8	20 ± 11.2
≥300	3 (4.5)	8.9 ± 0.4	143 ± 8	81 ± 10	62.3 ± 2.1	20.3 ± 5.7

All values are presented as means ± standard errors of the mean. Data was not significant at *p* > 0.05. N: Number of participants, HbA1c: Hemoglobin A1c, SBP: Systolic Blood Pressure, DBP: Diastolic Blood Pressure.

**Table 4:** Correlation between serum biomarkers HbA1c, SBP, and DBP with serum creatinine and eGFR.

Baseline Parameters	Serum Creatinine (mmHg)		eGFR (mL/min/1.73 m^2^)	
	Correlation, *r*	*p*-Value	Correlation, *r*	*p*-Value
AGE (µg/mL)	0.140	0.287	−0.145	0.269
sRAGE (pg/mL)	−0.199	0.121	0.185	0.151
Nε-CML (ng/mL)	0.31	0.015 *	−0.30	0.032 *
Cystatin C (ng/mL)	−0.238	0.061	0.164	0.199
HbA1c (%) ^†^	0.116	0.369	−0.049	0.704
SBP (mmHg) ^‡^	0.182	0.153	−0.126	0.325
DBP (mmHg) ^‡^	0.086	0.504	−0.017	0.893

Data controlled for HbA1c, SBP, DBP, and age. ^†^ Data controlled for SBP, DBP, age, and Nε-CML. ^‡^ Data controlled for HbA1c, age, and Nε-CML. * Significant at *p* < 0.05. AGE: Advanced Glycation Endproduct, sRAGE: soluble Receptor for AGE, Nε-CML: Nε-Carboxymethyllysine, HbA1c: Hemoglobin A1c, SBP: Systolic Blood Pressure, DBP: Diastolic Blood Pressure.

**Table 5:** Correlation between baseline parameters and Nε-CML.

Baseline Parameters	Nε-CML (ng/mL)	
	Correlation, *r*	*p*-Value
HbA1c (%)	−0.08	0.522
Age (years)	−0.20	0.107
Duration of diabetes (years)	0.06	0.625
AGE (µg/mL) ^‡^	0.500	0.000 *
sRAGE (pg/mL) ^‡^	−0.094	0.476
Cystatin C (ng/mL) ^‡^	−0.180	0.169

^‡^ Data controlled for HbA1c and age. * Significant at *p* < 0.05. AGE: Advanced Glycation Endproduct, sRAGE: soluble Receptor for AGE, Nε-CML: Nε-Carboxymethyllysine, HbA1c: Hemoglobin A1c.

**Table 6:** Simple and multiple logistic regression.

Baseline Parameters	Simple Logistic Regression			Multiple Logistic Regression^ a^		
	B	Crude OR (95% CI)	*p*-Value	B	Adjusted OR ^‡^ (95% CI)	*p*-Value
HbA1c (%)	0.132	1.141 (0.852, 1.527)	0.377			
SBP (mmHg)	0.006	0.730 (0.974, 1.039)	0.730			
DBP (mmHg)	0.033	1.033 (0.978, 1.092)	0.246 *			
AGE (µg/mL)	0.004	1.004 (1.000, 1.009)	0.049 *			
sRAGE (pg/mL)	0.001	1.001 (1.000, 1.002)	0.099 *			
Nε-CML (ng/mL)	0.357	1.429 (1.106, 1.845)	0.006 *	0.389	1.476 (1.112, 1.996)	0.008 **
Cystatin C (ng/mL)	0.000	1.000 (0.999, 1.000)	0.143 *			

* Significant at *p* < 0.250 on univariate analysis. ** Significant at *p* < 0.005 on multivariate analysis. ^a^ Forward Wald multiple logistic regression method applied. ^‡^ Adjusted for sRAGE. HbA1c: Hemoglobin A1c, SBP: Systolic Blood Pressure, DBP: Diastolic Blood Pressure, AGE: Advanced Glycation Endproduct, sRAGE: soluble Receptor for AGE, Nε-CML: Nε-Carboxymethyllysine.

**Table 7:** General characteristics and blood chemistry between placebo and Tocovid group.

General Characteristics	Placebo Group (*N* = 23)	Tocovid Group (*N* = 22)	*p*-Value
Gender			0.586
Male (%)	15 (65.2)	16 (72.7)	
Female (%)	8 (34.8)	6 (27.30	
Race			0.895
Malay (%)	14 (60.9)	12 (54.5)	
Chinese (%)	5 (21.7)	6 (27.3)	
Indian (%)	4 (17.4)	4 (18.2)	
Age (years)	63.3 ± 10.42	59.9 ± 10.24	0.283
Duration of DM (years)	17.9 ± 7.65	18.2 ± 10	0.893
HbA1c (%)	8.7 ± 1.5	9.0 ± 2	0.611
SBP (mmHg)	138.8 ± 15	136.2 ± 18.4	0.601
DBP (mmHg)	78.5 ± 9.4	77.0 ± 10.2	0.617
Weight (kg)	78.3 ± 12.8	78.2 ± 16.5	0.983
BMI (kg/m^2^)	29.3 ± 4.7	29.4 ± 5.4	0.978
Renal Parameters:			
UACR (mg/mmol)	128.7 ± 164.7	66.4 ± 61.8	0.101
Serum Creatinine (umol/L)	125.5 ± 56.6	120.2 ± 57.9	0.761
eGFR (mL/min/1.73 m²)	57.5 ± 25.1	63.1 ± 24.1	0.445
Biomarkers:			
AGE (µg/mL)	112.2 ± 149.5	136.1 ± 188.4	0.646
sRAGE (pg/mL)	1060.9 ± 438.8	1099.9 ± 408	0.759
Nε-CML (ng/mL)	2.4 ± 2.2	2.7 ± 2.5	0.695
Cystatin C (ng/mL)	1947.5 ± 1078	1941.3 ± 837	0.983
Safety Tests:			
Urea (mmol/L)	8.4 ± 4.7	6.3 ± 3.5	0.095
Total chol (mmol/L)	4.2 ± 0.9	4.6 ± 0.9	0.192
HDL (mmol/L)	1.1 ± 0.3	1.2 ± 0.2	0.728
AST (UI/L)	19.2 ± 6.9	25.4 ± 9.1	0.013 *
ALT (UI/L)	19.3 ± 11	29 ± 15.7	0.020 *

All values are presented as means ± standard deviations. * Data is significant (*p* < 0.05). N: Number of participants, HbA1c: Hemoglobin A1c, SBP: Systolic blood pressure, DBP: Diastolic blood pressure, BMI: Body mass index, DM: Diabetes mellitus, UACR: Urine albumin to creatinine ratio, eGFR: estimated Glomerular Filtration Rate, AGE: Advanced Glycation Endproduct, sRAGE: soluble Receptor for AGE, Nε-CML: Nε-Carboxymethyllysine, Total chol: Total cholesterol, HDL: High density lipoprotein, AST: Aspartate Aminotransferase, ALT: Alanine aminotransferase.

**Table 8:** Adjusted changes in metabolic, renal and biomarker parameters between placebo and Tocovid group.

Analytes	Placebo Group	Tocovid Group	Mean Difference	*p*-Value ^†^
HbA1c (%)	8.61 ± 0.17	8.45 ± 0.17	−0.16 ± 0.24	0.518
SBP (mmHg)	137.09 ± 2.81	130.48 ± 2.88	−6.62 ± 4.03	0.108
DBP (mmHg)	77.61 ± 1.78	77.91 ± 1.82	0.297 ± 2.57	0.909
Weight (kg)	78.22 ± 0.84	79.47 ± 0.86	1.24 ± 1.20	0.308
Renal parameters:				
UACR (mg/mmol)	66.93 ± 8.93	85.43 ± 9.14	18.45 ± 12.97	0.161
Sr Creatinine (μmol/L)	131.04 ± 2.92	119.76 ± 2.92	−11.28 ± 4.31	* 0.014 ^‡^
eGFR (mL/min/1.73 m²)	74.89 ± 11.51	63.57 ± 11.77	−11.31 ± 16.51	0.497
Serum biomarkers:				
AGE (µg/mL)	83.66 ± 27.20	89.82 ± 27.20	6.16 ± 38.50	0.874
sRAGE (pg/mL)	1088.32 ± 111.65	1246.36 ± 114.16	158.05 ± 159.77	0.328
Nε-CML (ng/mL)	2.56 ± 0.48	2.59 ± 0.49	0.28 ± 0.69	0.967
Cystatin C (ng/mL)	2172.45 ± 181.56	2031.99 ± 185.64	−140.46 ± 259.66	0.591
Safety tests:				
Urea (mmol/L)	7.33 ± 0.37	7.26 ± 0.38	−0.07 ± 0.54	0.896
Total chol (mmol/L)	4.52 ± 0.12	4.67 ± 0.12	0.15 ± 0.17	0.384
HDL (mmol/L)	1.17 ± 0.03	1.18 ± 0.03	0.01 ± 0.04	0.772
AST (IU/L)	21.21 ± 0.91	19.41 ± 0.93	−1.80 ± 1.35	0.190
ALT (IU/L)	26.45 ± 1.67	22.17 ± 1.71	−4.28 ± 2.46	0.089

All values are presented as means ± standard errors of the mean. ^†^ Data adjusted for baseline values and age. ^‡^ Data adjusted for baseline value, age, weight, HbA1c, SBP, DBP, age, weight, AGE, CML, sRAGE, and Cystatin C. * Significant at *p* < 0.05. HbA1c: Hemoglobin A1c, SBP: Systolic blood pressure, DBP: Diastolic blood pressure, BMI: Body mass index, DM: Diabetes mellitus, UACR: Urine albumin to creatinine ratio, eGFR: estimated Glomerular Filtration Rate, AGE: Advanced Glycation Endproduct, sRAGE: soluble Receptor for AGE, Nε-CML: Nε-Carboxymethyllysine, Total chol: Total cholesterol, HDL: High density lipoprotein, AST: Aspartate Aminotransferase, ALT: Alanine aminotransferase.
